# How Angular Mismatch and Surface Topography in Modular Head–Stem Taper Junctions in Total Hip Replacements Affects Fretting-Corrosion and Motion Under Uni-Axial Loading

**DOI:** 10.3390/s26113571

**Published:** 2026-06-04

**Authors:** Abigail Wade, Andrew Robert Beadling, Dominic Jones, Danielle De Villiers, Jo Cullum, Simon Collins, Michael George Bryant

**Affiliations:** 1 School of Mechanical Engineering, Institute of Functional Surfaces, University of Leeds, Leeds LS2 9JT, UK; 2 School of Engineering, University of Birmingham, Brimingham B15 2FG, UK; 3 MatOrtho^®^, Mole Business Park, Randalls Rd, Surrey KT22 7BA, UK

**Keywords:** total hip replacement (THR), taper, angular mismatch, fretting corrosion, micromotion, subsidence

## Abstract

Morse-type tapers at the head–stem junction in total hip replacements (THRs) provide many benefits to permit a successful surgical outcome. However, with the introduction of modular tapered devices comes complications associated with fluid ingress and motion at the interface that can cause fretting corrosion, which has been implicated in clinical failure. Increased surface roughness amplitude (R_a_) and angular mismatch to ensure taper contact closer to the equator of the femoral head are design features introduced for use with ceramic heads but have been adopted by metal head couples. While increased surface roughness amplitude has been found to contribute to fretting corrosion, there is a distinct lack of systematic studies investigating the interactions between angular mismatch and R_a_. This study measured the fretting corrosion and motion response of clinically representative samples, in part reference to ASTM F1875, when subjected to uniaxial incremental dynamic loading. The fretting corrosion response was measured in situ with an integrated three electrode electrochemical cell. Motion at the head–neck interface was measured with a bespoke motion measurement solution based on eddy-current principles which uses four sensors to allow motion to be fully characterised in three dimensions. Key findings from this study included a 5–10-fold increase in current measured in the increased roughness amplitude samples, suggesting an increased susceptibility to fretting corrosion without a corresponding increase in motion. The distal samples engaged around the opening of the taper interface and presented the lowest current measurements but most off-axis subsidence. Findings from this study indicate that optimisation of the taper interfaces in THR, in terms of fretting corrosion and motion, can be made and can be assessed using short-term preclinical tests.

## 1. Introduction

Modularity of the femoral component in total hip replacements (THRs) between the articulating head and femoral stem is facilitated by a Morse-type taper [1]. Introduced in the 1980’s as an alternative to mono-block femoral components, modular femoral components provided a number of benefits key to a successful surgical outcome, including permitting a different head material (typically cobalt chromium alloy or ceramic) to the stem (typically Co, Ti and Fe based alloys), intra-operative selection of various head sizes and offsets, and the ability to retain a well-fixed stem during revision surgery whilst allowing a change in head [2,3,4,5]. The 18th annual joint registry for England, Wales, Northern Ireland and Isle of Man (NJR) [6], reported that over half a million THR were implanted in the last six years, the vast majority including head–stem modularity. Whilst modularity addresses a number of key surgical limitations posed by mono-block femoral stems such as leg length inequalities, it does introduce additional interfaces for fluid ingress and motion for accelerated implant degradation.

The degradation process occurring at modular taper interfaces is complex and cannot be explained by one or even two basic corrosion and/or wear mechanisms. The mechanism most consistently supported by the literature describes a synergy between fretting and corrosion, i.e., fretting corrosion or mechanically assisted crevice corrosion [7]. This involves mechanical abrasion and de/repassivation of the 2–10 nm-thick surface oxide layer typically formed on passive biomedical alloys. This action, coupled with the taper geometry which forms a crevice, can initialise and sustain accelerated corrosion [7,8,9,10]. Wear and corrosion products created at the taper junction have been associated with adverse local tissue reactions, commonly presented in patients as pain followed by instability [11,12,13,14].

Recently, interest in the release of degradation products from the taper junction has been renewed owing to the higher than acceptable revisions rates of large diameter metal-on-metal total hip replacements [15]. However, according to the NJR [6], metal-on-metal bearing articulations form a very small percentage of those implanted today (e.g., MoM resurfacing with no modular taper) but adverse soft tissue reactions to particulate debris remains one of the more prominent reasons for revision, making up 13% of all revisions, of which the taper junction is one possible generation source. Additionally, medical device manufacturers are now required by European Union (EU) Medical Device Regulations (MDR) to demonstrate that the risk posed by these degradation products (i.e., nano-particulates) is biologically acceptable and as small as possible [16]. Modular taper degradation has also been reported for smaller diameter, other bearing couple and dual mobility system, indicating this phenomena is not just related to large diameter MoM THR.

Morse tapers are a cone-in-cone mechanical interference fit and were originally conceived to allow machine parts to be changed quickly without compromising torque transmission. Successful morse tapers were originally designed to be highly conforming, smooth, hard, long, and with a slight cone/taper angle [17,18,19]. All of these design features are primarily to ensure a sufficient interference fit, preventing motion that might affect the quality of the workpiece and cause damage to the tapered surface [1,20]. Arguably, the most important tolerance is that of the taper angle to ensure a tight uniform fit between male and female tapered surfaces. Tolerances detailed by ISO 1947 [19] describes twelve different taper angle tolerance grades with the tightest being AT1 with 10″ (or 0.003°) and the loosest being AT12 with 21′38″ (or 0.36°) for a taper length of 10–16 mm. Most modern CNC machines have tapered interfaces that are made to AT3 or tighter [20,21]. Although manufacturing tolerances of taper angles in THR are not public knowledge, published measurements of THR tapers from the same manufacturer would indicate a tolerance closer to that of around 0.05° falling into the AT8 grade [19,22,23]. Compared to tapered junctions used in industrial/cutting applications, head–stem tapers used in THR tend to be shorter, and with a greater taper angle than machine morse tapers. They also often present a threaded type surface finish and greater levels of angular mismatch (i.e., female minus male taper angle) for engagement at specific contact regions rather than over the whole surface [1,24]. A threaded surface finish to increase the roughness amplitude and a proximal angular mismatch (i.e., contact at the inner most region of the head taper) was introduced for use with ceramic heads to reduce the risk of burst fracture [1]. However, these design parameters appear to have been translated over to metal head couples. The problem of taper degradation in THR is further complicated by the biomechanical loading profile which is complex, cyclic in nature, and often off-axis [25]. Morse tapers were designed to transmit high torques under a dominant compressive axial load [1]. The sorts of mechanical loads experienced at the taper junction include loading in six axes and can exceed body weight by almost a factor of four [25,26,27]. The complex biomechanical loading facilitates motion and fluid ingress with an abundance electrochemically active species available in the body [7].

There is a wide variation in clinical head–stem tapers with over thirty different design variations in terms of material combination, geometry and surface topography and no current standardisation [1]. As such, two tapers of the apparent same type (e.g., 12/14) are not uniform and can present different surface topographies, taper length, opening diameter and taper angle [22,23,28]. Performance of the taper junction has been found to vary with different designs parameters [27,29,30,31]. Evidence relating taper design to clinical performance, however, is often inconclusive, conflicting or limited to high-level descriptions, e.g., long ‘smooth’ tapers tend to perform better than short ‘rough’ tapers [32,33,34]. In vitro experimental studies have attempted to investigate the effects of individual design factors on performance albeit with conflicting results [31,35,36]. For example, Ouellette et al. [29] reported little to no correlation between surface topography and fretting corrosion/motion subject to uni-axial loading, whereas Panagiotidou et al. [37] noted evidence of oxide disruption with threaded ‘rough’ tapers but not on the investigated ‘smooth’ tapers. The clinical implication of manufacturing tolerances and engagement mismatches on fretting corrosion of modular taper devices is still to be fully elucidated. In silico studies report increased contact stress, motion and predicted wear with an increase in angular mismatch [38,39,40]. Additionally, there was a difference in observed behaviour depending on if the angular mismatch was positive or negative, i.e., resulted in a ‘proximal’ engagement (contact at the top of the male taper) or ‘distal’ engagement (contact at the taper opening). Distal engagements were reported to be present a smaller gap at the taper which is thought to help resist corrosion related degradation [41]. Most experimental studies employ the use of cobalt chromium alloy (CoCrMo) heads and titanium alloy male taper components but within the UK over 62% of primary stems implanted in 2020 were cemented, of which over 70% are products manufactured from a high-nitrogen stainless steel alloy [6]; thus, research findings may be somewhat unrepresentative of the clinical situation. Metal heads are still the most commonly implanted with an increasing trend of ceramic heads, indicating that the most common head–stem material couple in the UK is CoCrMo-stainless steel. Without a doubt, the degradation processes occurring are complex and multifactorial and understanding how different parameters affect the complex web of degradation processes is considered important. Insufficient consideration for the different aspects of degradation and how they may interact is a possible reason for the lack of common understanding and sometimes conflicting findings within literature.

Generally, there is a lack of systematic controlled experimental studies that have investigated the effect of angular mismatch, a key manufacturing tolerance, and how this might interact with surface topography within stainless steel–CoCrMo modular taper interfaces. The aim of this study was to conduct a systematic experimental investigation into the effect of the angular mismatch and surface topography present in clinically available THR on the fretting corrosion and motion response through the implementation of a combined fretting-corrosion and micromotion sensing preclinical testing framework. Outputs from this study are used to help understand how angular mismatch and surface topography affects the performance of the taper junction.

## 2. Materials and Methods

The short-term performance of taper junctions was studied in part-reference to ASTM F1875-08 [42]. This included measuring the fretting corrosion and motion response of the taper interfaces subject to incremental uniaxial loading, with peak loads between 0.5 and 4 kN, to replicate the principle joint reaction forces seen in-vivo [25]. The fretting corrosion response was measured using electrochemical potentiostatic measurements. Relative motion at the taper was measured using a bespoke sensor and protocols.

### 2.1. Samples

Clinically representative ‘12/14’ taper samples were manufactured, cleaned and packaged by MatOrtho Ltd. (Leatherhead, UK). The male taper samples were made from High Nitrogen Stainless Steel according to ISO 5832-9 [43]. The head samples were manufactured from Cobalt Chromium alloy (CoCrMo) according to ISO 5832-6 [44] with + 0 mm offset and 28 mm head diameter. These materials were selected to represent the most common head and stem material combination currently implanted in the UK [6]. Samples were manufactured and cleaned using industry standard processes and surface topographies were created as part of the CNC lathe process.

To investigate the effect of angular mismatch and surface topography on the corrosion and motion of modular taper interfaces, six different test groups were created to demonstrate the range of that seen clinically [23]. This included three different engagement groups and two surface finishes for six sample groups (see Table 1). The three engagement groups included: distal engagement, with contact at the opening of the taper interface; matched engagement, where contact was assumed to be distributed across the interface; and proximal engagement, with contact towards the inner most portion of the head taper interface. The three different engagement groups were created by design by varying the female taper angle relative to the male taper to create a controlled and repeatable angular mismatch. Angular mismatch was calculated using the same measurement protocol developed previously by the authors [23]. This included measuring the samples using a coordinate measurement machine (CMM, Legex 322, Mitutoyo, Sakado, Japan), calculating and subtracting the female taper cone angle from the male. Table 1 details the angular mismatch for each of the sample groups. The matched and proximal engagement groups were representative of those presented by clinically available THR [23]. The distal mismatch was exaggerated by approximately −0.05° to ensure a distal mismatch was able to be achieved in practice.

Two different male taper surface topographies were studied, ‘rough’ and ‘smooth’, both of which were manufactured by lathe processing. Table 1 shows example roughness profiles of both a ‘rough’ and ‘smooth’ surface finish with a 2 mm surface topography sample length. Surface topography was measured using a vertical scanning interferometer (VSI, NPFlexTM, Bruker, Billerica, MA, USA); details of the analysis can be found in Section 2.6. Surface roughness within the female taper was as machined.

### 2.2. Modular Taper Assembly and Disassembly

The female and male components were assembled quasistatically, at a rate of 0.04 mms^−1^ to 2 kN in dry conditions using a uniaxial material testing machine (Instron 3869, Norwood, MA, USA). Bespoke precision manufactured fixtures were used to control the assembly process beyond that according to ISO 7206-10 [45]. ISO 7206-10 [45] specifies a loading tolerance alignment of 0 ± 1° with the longitudinal axis of the male taper axis and the female taper is uncontrolled. Whereas the methodology used to assemble these samples included holding the loading, female taper and male taper axis concentric to a tolerance of ± 0.001°. Further details of the assembly and disassembly procedure can be found in Wade et al. [46].

### 2.3. Uniaxial Dynamic Loading

**Figure 1:**
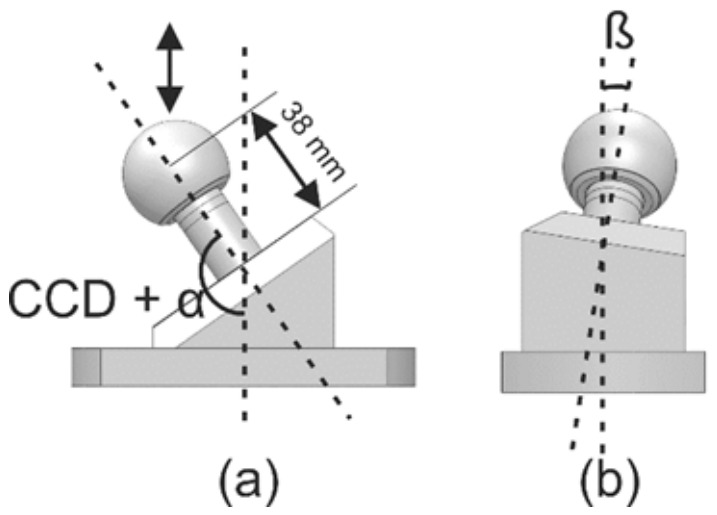
Illustration of the fixtures used for incremental dynamic loading showing the (**a**) alpha, CCD and (**b**) beta angles.

Uniaxial dynamic loading was undertaken with the samples in an anatomical orientation according to ISO 7206-4 [47] (Figure 1a). It describes an alpha and beta angle of 10 and 9°, respectively, along with an assumed centrum–collum–diaphyseal angle (CCD) of 135° to the loading axis (Figure 1a,b). ISO 7206-4 [47] also describes that the head offset should be reported; this study used a head offset of approximately 38 mm. This was aimed to replicate that used by clinically available THR whose head offsets tended to range from 27 to 40 mm [48]. Figure 1 shows an illustration of the fixtures that were made of stainless steel.

The loading sequence was applied using a dynamic material testing machined (Instron E10 000, Norwood, MA, USA) and consisted of a sinusoidal waveform starting with a peak-to-trough range from 5 N to 500 N. The wave was applied at 1 Hz, for 600 cycles and followed by a 10 min hold phase for the fretting corrosion tests and a 10 s hold phase for the motion tests. During the hold phase, the load was held at half the peak force of the preceding increment. This was repeated for a further 7 stages, increasing the peak height of the sine wave by 500 N until the final increment of 4000 N, resulting in a total of 8 increments.

### 2.4. Fretting Corrosion Protocol

**Figure 2:**
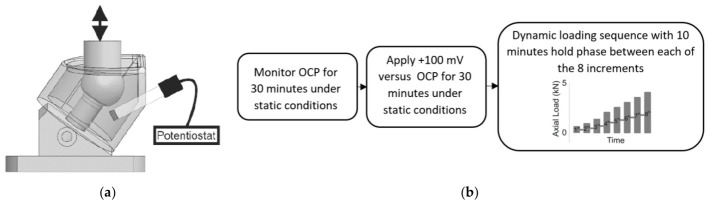
(**a**) Illustration of the integrated fretting corrosion cell and (**b**) flow chart of the electrochemical measurements taken throughout a single test.

The fretting corrosion test setup consisted of a three-electrode electrochemical cell, integrated into the test arrangement (Figure 2) to facilitate real-time measurement of corrosion in situ. A bath was mounted on the male component and the taper junction (the only metallic interface exposed to the electrolyte) was immersed in 100 mL of phosphate buffered saline (PBS) solution. The implant sample acted as the working electrode. An Ag/AgCl reference electrode and platinum disc counter electrode completed the electrochemical cell. The electrodes were connected to a floating ground potentiostat (IVIUM Compactstat, Eindhoven, The Netherlands). Figure 2a,b show the electrochemical and loading regime applied in this study.

Prior to dynamic loading, the Open Circuit Potential (OCP) of the system was allowed to equilibrise for 30 min under static conditions in the absence of an applied normal load. A potentiostatic technique was applied to provide a quantitative measure of the net electrochemical currents owing to corrosion. A +100 mV vs. OCP under static conditions was then applied for a further 30 min, before starting the dynamic loading sequence. This was to allow the measured current to settle to a sufficiently low ampere (around 500 nA), before the application of the dynamic loading sequence (Figure 2b). The overpotential was selected to force the anodic reaction away from the equilibrium to allow quantitative measurement of net current, without forcing it too far, so as to cause changes in the oxide layer that will no longer be representative. This was shown by current measurements settling to very low values during static phases, in the region of 100 to 500 nA.

**Figure 3:**
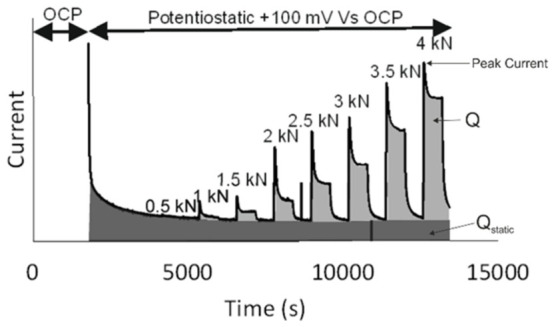
Schematic of a typical anodic current transient where an increase in current is associated with fracture of the passive oxide film during loading. The area under the current-time graphs was calculated to obtain charge, where the dark grey shading corresponds to ion transfer due to static corrosion and the light grey regions to tribocorrosion mechanisms. Net corrosion current was typically in the μA range.

Current was measured at a frequency of 1 Hz which provided information on the passivation and depassivation behaviour of the tapered interface. Figure 3 shows a typical anodic current transient demonstrating how under dynamic loading an increase in current corresponds to the depassivation due to abrasion and subsequent repassivation of the oxide layer, causing a measurable increase in current across the working and counter electrodes. The measured current reflects the electrochemical reaction rate at the taper interface under the imposed potential. Low, steady current suggests a passive film. Fretting disrupts the film, exposes fresh metal and produces current transients as depassivation and repassivation occur. Integrating current over time gives cumulative charge (Q), a comparative metric of overall electrochemical activity during the loading history. Faraday’s law can convert Q to an equivalent metal mass (m=(QM)/(nF), where M is molar mass, n is the number of electrons per dissolved metal ion, and F is Faraday’s constant), but for passive, multi-component alloys the current also contains film-growth and cathodic contributions, so the conversion is uncertain. A greater increase in current would indicate a greater amount of oxide layer disruption. This study compared total charge transfer (Q), peak current and average current measured for each increment, after removing a baseline associated with static corrosion (dark grey shaded regions shown in Figure 3, Q_static_). The baseline current was calculated using a line of best fit from the data points taken prior to loading and the last two minutes of each hold phase. Charge was calculated as it is considered to be directly proportional to material loss due to corrosion according to Faraday’s law. Charge transfer due to tribocorrosion (Q, light grey shaded regions shown in Figure 3) was calculated by integrating current with respect to time and subtracting the baseline charge (Q_static_, darker grey shaded region in Figure 3).

### 2.5. Motion Arrangement and Protocol

**Figure 4:**
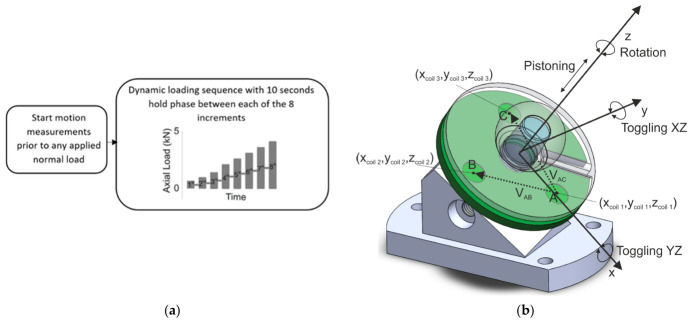
Schematic of (**a**) motion measurement protocol and (**b**) coil board-target configuration with annotated motions directions.

Custom motion sensors were developed, using the principle of eddy-current formation as the transducer mechanism between sensing coil and conductive target. This was used to sense the relative motion between the head and stem in situ (Figure 4). Motion at the taper junction was measured at a frequency of 100 Hz during the incremental dynamic loading methodology detailed in Section 2.3 (Figure 4a). To capture all types of motion at the taper interface, four sensing coils were used, as shown in Figure 4b. This included separating the motions into axial motions along the taper axis (pistoning), off-axis motions in two dimensions (toggling YX and toggling XZ) and rotational about the taper axis (rotation). Details of the developed sensing solution, calibration and how the different motions were calculated can be found in the Supplementary Data document.

**Figure 5:**
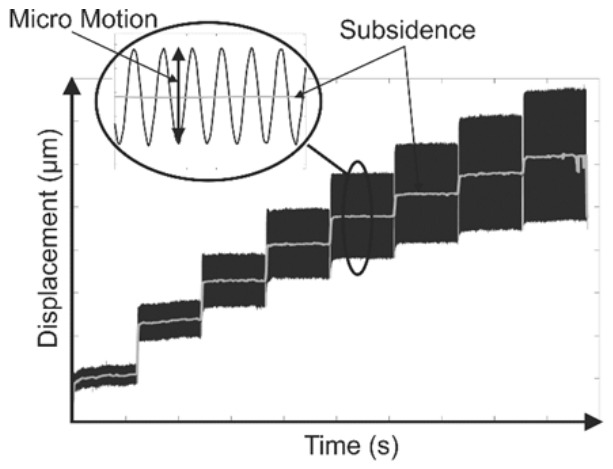
Schematic of subsidence (light grey line) and micro motion (dark grey line), evidence of the 8 loading increments can be seen with a short 10 s hold phase between each.

In addition to separating motion into pistoning, toggling YZ, toggling XZ and rotation, motion was further divided into subsidence and micro motion. Subsidence was attributed to how the heads moved further down into the taper over the course of the experiment, while micro motion was attributed to the small displacements about subsidence. Figure 5 shows a schematic of pistoning motion over the eight loading increments, where subsidence is shown by the light grey line following the overall form of the motion measurements, and micro motion is the higher-frequency (1 Hz) motion. Subsidence at each increment was calculated as the average subsidence displacement over a given increment. Likewise, micro motion was calculated as the average peak-to-trough height of the smaller motions about subsidence over a given increment.

The average subsidence and micro motion measured at each increment contained both elastic deformation of the components and rigid body motion at the taper junction. To isolate the amount of relative motion occurring at the tapered junction, motion measurements were performed on an equivalent monoblock sample manufactured by MatOrtho Ltd. (Leatherhead, UK). The monoblock was a sample that was a surplus ‘smooth’ matched sample that was assembled to a high force of 8 kN and welded at the taper opening. Subsidence and micro motion measured from the monoblock were then subtracted from that measured on each sample. Measurements of the equivalent monoblock can be found in the Supplementary Data document. The same samples used for fretting corrosion tests were used for motion assessment following the controlled disassembly process. Surfaces were inspected for signs of damage, sonicated cleaning in acetone, air dried and reassembled to 2 kN.

### 2.6. Surface Analysis

**Figure 6:**
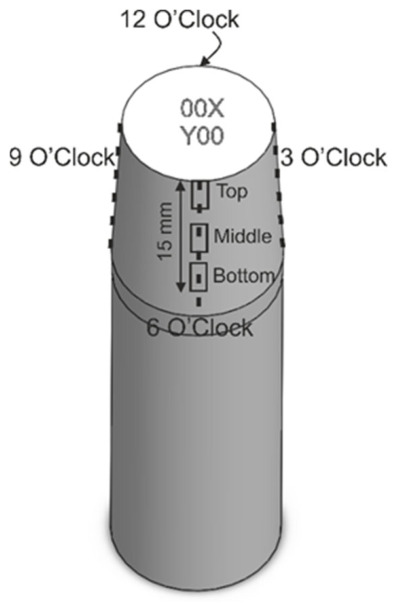
Location of the twelve equally spaced VSI scans relative to the male taper component geometry.

Surface topography measurements of the male taper components were taken before and after testing using a vertical scanning interferometer (VSI, NPFlexTM, Bruker, San Jose, CA, USA). Twelve equally spaced scans of the male tapers were carried out with a 20 × magnification, over an area of a 2 mm by 0.5 mm rectangle. Three scans toward the top, middle and bottom of the taper interface were taken in the 12, 3, 6 and 9 o’clock locations around the male taper component, shown schematically in Figure 6. The scan toward the top of the taper starts at the very top of the nominal engagement area and the end of the bottom scan ends 15 mm below that to capture any changes to the surface due to engagement with the female taper.

These scans were then analysed using Vision64 (Bruker, San Jose, CA, USA). Roughness measurements were performed with a cut-off length of 0.8 mm according to ISO 4288 [49]. Table 2 summarises the roughness parameters used to describe any changes to the surface topography in terms of amplitude (S_a_ and S_k_) and distribution of protruding peaks (S_pd_). These were presented as averages over the whole surface while spatial variation of surface roughness was compared as an indication of localised changes to the surface topography due to interface contact and degradation.

### 2.7. Statistics

All data are presented as mean ± one standard deviation. There were three samples of ‘smooth’ distal, matched and proximal samples, and two samples of ‘rough’ distal, matched and proximal samples, allowing for three and two repeats, respectively. Statistical comparison of results between the different engagement groups with the ’smooth’ male taper topography was achieved using a two-tailed paired Students *t*-test. The level of significance was set at a *p*-value of 0.05. Analysis was performed using Excel (Microsoft, Redmond, WA, USA).

## 3. Results

### 3.1. Fretting Corrosion

**Figure 7:**
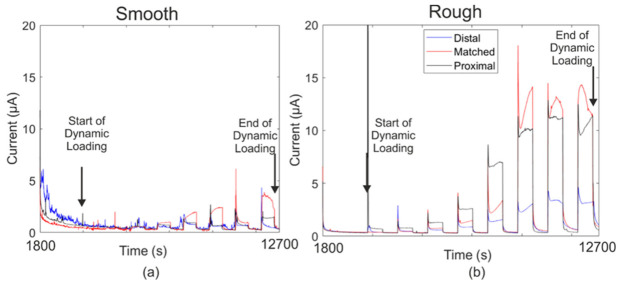
Example current transients of the (**a**) ‘smooth’ and (**b**) ‘rough’ samples.

The anodic current transient response for modular taper components is shown in Figure 7. After stabilisation of the OCP and application of the applied electrochemical overpotential, a decay in the net anodic current to a stable baseline value was observed. Upon the application of cyclic loading, all samples presented evidence of passive oxide layer disruption indicated by a sudden and sustained increase in current. The magnitude of current increased with increasing loading increment and recovered to the baseline during hold phases. The current signal differed between the six samples groups.

Comparing between the ‘smooth’ and ‘rough’ samples of equivalent engagements, the ‘rough’ samples presented greater currents compared to the ‘smooth’. The ‘rough’ samples also presented a more consistent spike in current upon the onset of a loading increment (Figure 7b) compared to the ‘smooth’ (Figure 7a), which was less consistent. For example, the ‘smooth’ matched sample in Figure 7b presented increasing current trends in the fourth to sixth increment followed by a spike and reduction in current in the seventh increment.

Comparing between the different engagement groups of equivalent surface topographies, the distal samples presented the smallest currents, while the matched presented the greatest. Within the ‘smooth’ samples, a variation in the recorded current signal was also noted. The ‘smooth’ distal samples presented a nosier signal compared to the proximal.

**Figure 8:**
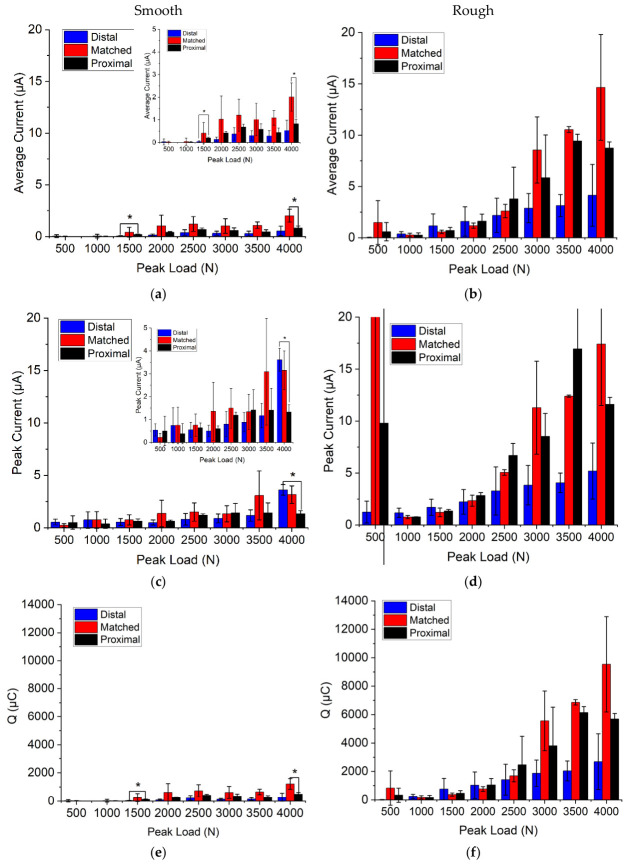
Fretting corrosion results showing: average current per increment for the (**a**) ‘smooth’ samples and (**b**) ‘rough’ samples, peak current per loading increment for the (**c**) ‘smooth’ samples and (**d**) ‘rough’ samples, and charge transferred per loading increment for the (**e**) ‘smooth’ samples and (**f**) ‘rough’ samples. * indicates significant difference with *p*-value < 0.05.

The average current, peak current and total charge transfer for all samples at each loading increment is shown in Figure 8. Comparing between the ‘rough’ and ‘smooth’ samples, the ‘rough’ presented a greater average current (Figure 8a versus Figure 8b), peak current (Figure 8c versus Figure 8d) and charge (Figure 8e versus Figure 8f) compared to the ‘smooth’ for a given engagement. This was most evident at higher loading increments. The greatest difference between the ‘rough’ and ‘smooth’ samples was seen in the matched engagement group. In the final increment, the ‘rough’ matched sample presented a 12.6 ± 5 µA greater average current and 8318 ± 3351 µC greater charge compared to the ‘smooth’ matched (five-fold increase). The distal engagement group presented the smallest difference between ‘rough’ and ‘smooth’. In the final loading increment, the ‘rough’ distal sample presented a greater average current of 3.6 ± 2.1 µA and charge 2413 ± 1960 µC compared to the ‘smooth’ distal (six-fold increase). Likewise, the ‘rough’ proximal presented a greater average current of 8.0 ± 0.5 µA and charge of 5196 ± 459 µC compared to the ‘smooth’ proximal in the final loading increment (10-fold increase).

Within the ‘smooth’ samples, the matched group tended to present greater average current (Figure 8a) and charge (Figure 8e) per increment than the proximal or distal groups, most evident at higher loading increments with peak force greater than 2000 N. Statistically greater average current and charge was found between the matched and proximal groups in the final increment (2.0 ± 0.6 µA versus 0.8 ± 0.2 µA, *p*-value < 0.05). The matched also presented a greater current and charge compared to the distal but this was not found to be statistically significant (2.0 ± 0.6 µA versus 0.5 ± 0.4 µA, *p*-value of 0.07). The distal samples presented the lowest average current and charge compared to the matched or proximal. This was not the case for peak current (Figure 8c). The proximal samples presented the lowest peak current in the final increment, compared to the matched (1.3 ± 0.3 µA versus 3.1 ± 0.8 µA, *p*-value < 0.05) or distal (1.3 ± 0.3 µA versus 3.6 ± 0.5 µA).

When comparing between the different engagement groups in the ‘rough’ samples, similar patterns can be seen as was with the ‘smooth’. More specifically, the distal samples presented the smallest average current (Figure 8b) and charge (Figure 8f) and matched the greatest. In this case, however, the distal samples also presented the smallest peak current (Figure 8d) compared to the matched or proximal. In the final increment, the distal presented an average current of 4.1 ± 3.0 µA, matched 14.7 ± 5.1 µA and proximal 8.7 ± 0.6 µA. The peak current (Figure 8d) found in the ‘rough’ samples presented similar trends to the average current.

### 3.2. Motion

#### 3.2.1. Subsidence

**Figure 9:**
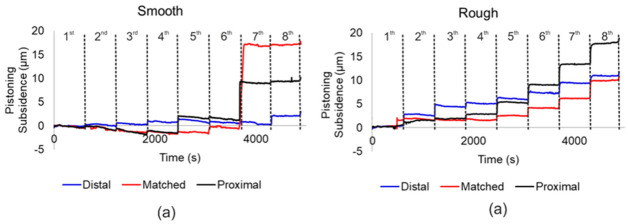
Example of pistoning subsidence for the (**a**) ‘smooth’ and (**b**) ‘rough’ samples with annotations indicating the boundary of each increment. Refer to Figure 4 and Section 2.5 for subsidence descriptions.

Figure 9a,b show the pistoning subsidence displacement (i.e., the head migrating axially down the male taper) of the six different sample groups for ‘rough’ and ‘smooth’ samples, respectively. Subsidence tended to increase with increasing loading increments, indicating that the heads were still seating. The ‘smooth’ samples tended to present evidence of sudden subsidence shown in Figure 9a by the ‘smooth’ matched and proximal samples upon the onset of the seventh loading increment with peak axial force of 3.5 kN. The ‘smooth’ proximal and matched samples also presented evidence of the heads moving back up the male taper before the sudden seating event. This was not observed by the ‘smooth’ distal sample that presented minimal pistoning subsidence compared to the ’smooth’ matched or proximal (Figure 9a). The ‘rough’ samples (Figure 9b) tended to migrate down the taper axis more gradually than the ‘smooth’ shown by the more step-wise increase with each increment.

**Figure 10:**
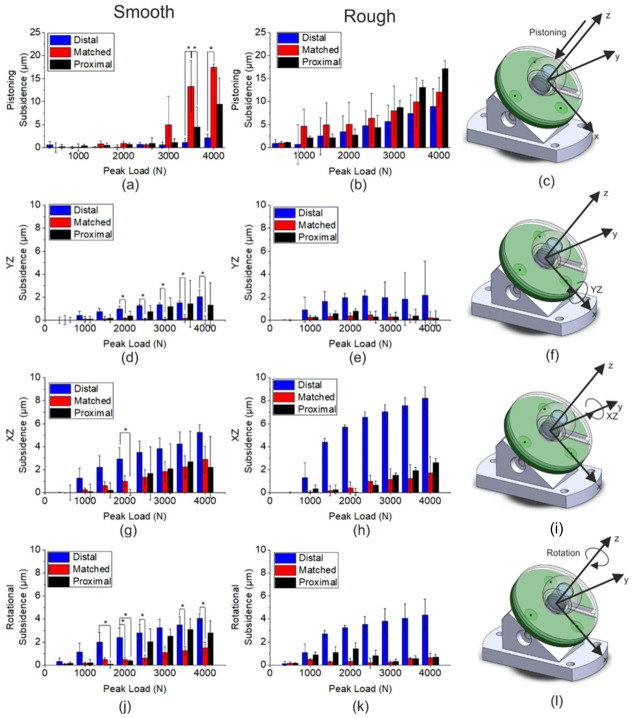
Magnitude of subsidence at each loading increment for the (**a**,**d**,**g**,**j**) ‘smooth’ and (**b**,**e**,**h**,**k**) ‘rough’ samples, in each of the different directions: (**a**,**b**) pistoning, (**d**,**e**) YZ, (**g**,**h**) XZ and (**j**,**k**) rotation shown schematically in adjacent subfigures (**c**,**f**,**i**,**l**). Significant difference between the different engagement groups of the ‘smooth’ samples is indicated by ‘*’.

Figure 10 shows the average subsidence of the femoral head relative to the male taper in the pistoning (axial), YZ, XZ and rotational directions during cyclic loading. In line with the greater loading vectors acting along the taper axis and bending moments in the XZ plane due to sample orientation (Figure 4b), the greatest magnitude of subsidence tended to be in the pistoning direction (Figure 10a,b). Subsidence in the XZ (Figure 10g,h) direction presented the second largest values of subsidence followed by rotation (Figure 10j,k) and YZ subsidence (Figure 10d,e).

Comparing between the ‘smooth’ and ‘rough’ samples of equivalent engagement, the ‘smooth’ matched and proximal samples present larger increases in pistoning subsidence (Figure 10a) in the final load increments. Additionally, the ‘rough’ matched and proximal samples (Figure 10b) presented a more gradual increase with each loading increment. The sudden subsidence events in the ‘smooth’ matched samples resulted in a greater magnitude of pistoning subsidence in the final increment at peak force of 4 kN compared to the ‘rough’ matched (17.4 ± 0.7 µm versus 12.0 ± 3.2 µm). This was not the case for the distal or proximal engagements, where the ‘rough’ samples presented a greater magnitude of subsidence compared to the ‘smooth’ (8.9 ± 3.9 µm versus 2.1 ± 0.9 µm distal engagement and 17.2 ± 1.6 µm versus 9.5 ± 5.6 µm proximal engagement).

Differences between the ‘smooth’ and ‘rough’ samples were also seen in the XZ (Figure 10d,e) and rotational directions (Figure 10j,k). The ‘rough’ distal samples presented a greater amount of XZ subsidence compared to the ‘smooth’ distal, presenting subsidence of 8.2 ± 1.0 µm versus 5.3 ± 0.6 µm in the final loading increment. In the rotational direction, the ‘smooth’ matched and proximal samples presented a greater amount of subsidence compared to the ‘rough’ matched and proximal. In the final increment, the ‘smooth’ matched and proximal samples presented subsidence of 1.5 ± 0.5 µm and 2.8 ± 1.0 µm compared to the ‘rough’ proximal and matched samples with 0.7 ± 0.3 µm and 0.7 ± 0.2 µm, respectively. Unlike pistoning, subsidence in the XZ, YZ and rotational direction tended to increase proportionally with each increment as opposed to discrete subsidence events at particular loading increments.

Within the ‘smooth’ samples there was a difference in subsidence behaviour between the different engagement groups. The distal samples tended to present the smallest magnitudes of pistoning subsidence compared to the matched or proximal (Figure 10a) but larger values in the remaining three motions, i.e., YZ (Figure 10d), XZ (Figure 10g) and rotation (Figure 10j). In the final loading increment (Figure 10a), the distal sample presented a pistoning subsidence of 2.1 ± 1.0 µm compared to the matched with 17.4 ± 0.7 µm (*p*-value < 0.05) and proximal 9.5 ± 5.6 µm (*p*-value > 0.05). In contrast, when comparing rotational subsidence in the final increment, distal presented a subsidence of 4.1 ± 0.4 µm compared to matched with 1.5 ± 0.5 µm (*p*-value < 0.05) and proximal with 2.8 ± 1.0 µm (*p*-value > 0.05). Likewise, in the YZ direction in the final loading increment, distal presented a subsidence of 2.0 ± 0.6 µm compared to matched with 0.0 ± 0.4 µm (*p*-value < 0.05) and proximal with 1.3 ± 2.0 µm (*p*-value > 0.05).

Comparing the different engagement groups in the ‘rough’ samples, similar trends were seen as with the ‘smooth’. That being the distal samples presented the smallest ‘pistoning’ subsidence but greater off-axis and rotational subsidence. In the final loading increment, the distal samples presented an XZ subsidence of 8.2 ± 1.0 µm compared to the matched with 1.7 ± 1.2 µm and proximal with 2.6 ± 0.4 µm. Likewise, in the rotational direction, the distal present subsidence of 4.4 ± 1.4 µm in the final increment compared to the matched with 0.7 ± 0.3 µm and distal 0.7 ± 0.2 µm.

#### 3.2.2. Micro Motion

**Figure 11:**
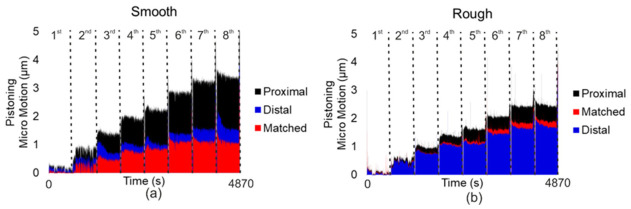
Example area plot of pistoning micro motion of the (**a**) ‘smooth’ and (**b**) ‘rough’ samples on a cycle-by-cycle basis with annotated increment boundaries.

Like subsidence, the magnitude of micro motion increased with increasing loading increment. This is shown in Figure 11a,b which presents area plots of pistoning micro motion on a cycle-by-cycle basis for a typical example of each of the six sample groups. Almost all samples at each increment presented a tendency for micro motion to peak upon the onset of loading and then settle, more evident in some cases than others, such as the smooth distal sample in the 8th increment (peak force of 4 kN) (Figure 11a). Comparison between Figure 11a,b also suggests differences between the different samples groups. The ‘smooth’ proximal samples (Figure 11a) demonstrated a greater magnitude compared to the ‘smooth’ distal and matched. The ‘rough’ samples tended to present less of a difference between the different engagement groups (Figure 11b).

**Figure 12:**
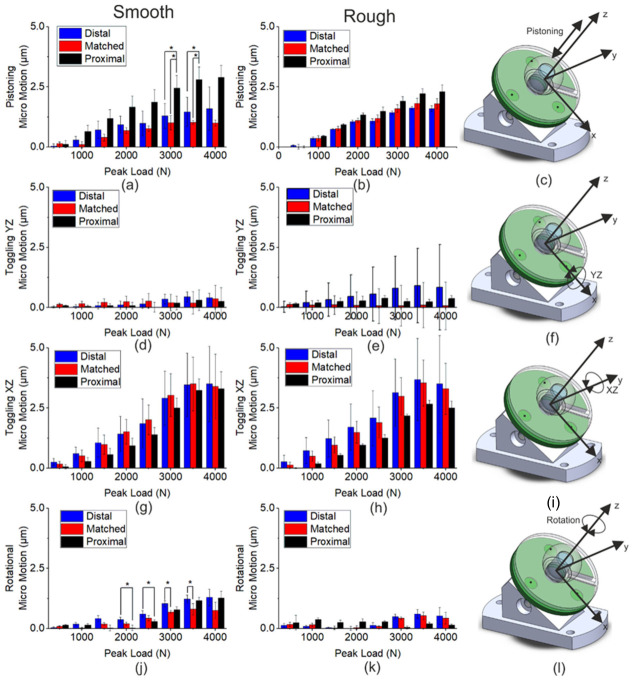
Micro motion at each loading increment for the (**a**,**d**,**g**,**j**) ‘smooth’ and (**b**,**e**,**h**,**k**) ‘rough’ samples, in each of the different directions: (**a**,**b**) pistoning, (**d**,**e**) YZ, (**g**,**h**) XZ and (**j**,**k**) rotation shown schematically in adjacent subfigures (**c**,**f**,**i**,**l**). Significant difference between the different engagement groups of the ‘smooth’ samples is indicated by ‘*’.

Micro motion presented an increasing trend with loading increment in each of the different directions as shown in Figure 12. The greatest magnitude of micro motion was shown in the pistoning and togging XZ direction, whereas toggling YZ and rotational micro motion presented the smallest magnitudes.

Comparing the ‘smooth’ and ‘rough’ samples for a given engagement, similar magnitudes of micro motion were observed for a given engagement. In the pistoning direction (Figure 12a,b), the matched engagement group presented the greatest difference, with the ‘rough’ matched presenting a greater magnitude of 0.8 ± 0.2 µm in the final increment. Smaller differences were found between the distal (1.6 ± 0.9 µm ‘smooth’ versus 1.6 ± 0.2 µm rough, in the final increment) and proximal (2.9 ± 0.5 µm versus 2.3 ± 0.3 µm in the final increment) engagement groups. Like pistoning, toggling XZ presented small differences between the ‘smooth’ and ‘rough’ samples of equivalent engagement (Figure 12g,h). The greatest differences were seen in the proximal group, where the ‘smooth’ proximal presented a greater average micro motion (3.3 ± 0.7 µm) in the final loading increment compared to the ‘rough’ proximal (2.5 ± 0.3 µm). Only small magnitudes of micro motion were recorded in the toggling YZ and rotational direction. One difference noted in toggling YZ direction was that the ‘rough’ distal engagement group presented a much greater variability compared to the ‘smooth’ distal. The ‘smooth’ samples appeared to present a greater amount of rotational micro motion compared to the ‘rough’, more evident in the distal (by 0.8 ± 0.3 µm in the 8th increment) and proximal engagement groups (by 1.1 ± 0.3 µm in the 8th increment) (Figure 12j,k).

Comparing the different engagements in the ‘smooth’ samples, the proximal samples tended to present a greater magnitude of pistoning micro motion compared to the distal and matched with significant differences in the 6th (2.5 kN peak axial force) and 7th (3 kN peak axial force) loading increments (Figure 12a). In the seventh loading increment, the proximal samples presented a magnitude of 2.8 ± 0.5 µm compared to the distal with 1.5 ± 0.6 µm (*p*-value < 0.05) and matched with 1.0 ± 0.2 µm (*p*-value < 0.05). In the rotational direction, the matched presented the smallest level of micro motion compared to the distal and proximal. A significant difference was found between the distal and matched samples in sixth and seventh loading increments, wherein only a marginal difference of 0.5 ± 0.2 µm in the sixth increment was found. Little difference was seen between the different engagement groups in the toggling directions.

Comparing within the ‘rough’ samples, in the pistoning direction (Figure 12b), the proximal samples presented the greatest average micro motion compared to the distal or matched. In the final increment, the proximal samples presented a magnitude of 2.3 ± 0.3 µm compared to the distal with 1.6 ± 0.2 µm and matched with 1.8 ± 0.2 µm. In contrast, the proximal samples on average presented less toggling XZ micro motion compared to the distal or matched samples (2.5 ± 0.3 µm versus 3.5 ± 2.0 µm and 3.3 ± 1.1 µm, Figure 12h).

### 3.3. Surface Analysis Pre and Post Testing

**Figure 13:**
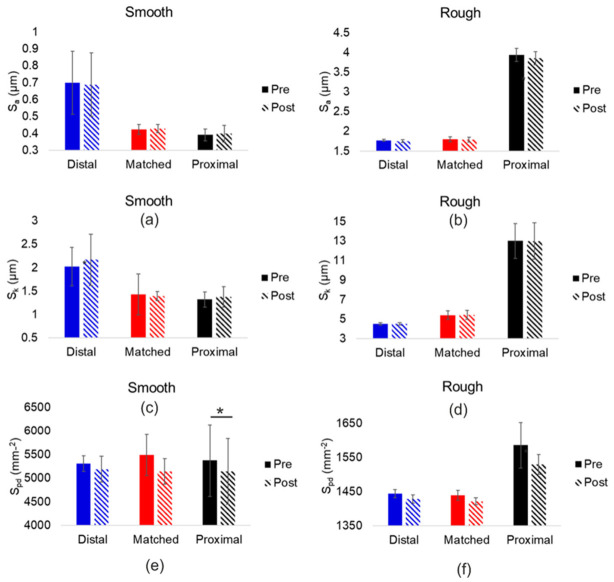
Surface topography of the as manufactured surface (pre) compared to post testing: (**a**) S_a_ of the ‘smooth’ samples, (**b**) S_a_ of the ‘rough’ samples, (**c**) S_k_ of the ‘smooth’ samples, (**d**) S_k_ of the ‘rough’ samples, (**e**) S_pd_ of the ‘smooth’ samples and (**f**) S_pd_ of the ‘rough’ samples. Statistical difference between pre and post testing was indicated by an asterisks, *p*-value < 0.05.

Figure 13 shows the average surface topography parameters pre and post testing measured using VSI. The as-manufactured ‘rough’ samples presented a greater roughness amplitude than the ‘smooth’ (S_a_ = 2.47 ± 1.02 µm versus 0.51 ± 0.17 µm, Figure 13a,b). Likewise, the ‘rough’ presented a greater S_k_ of 7.67 ± 3.80 µm versus the ‘smooth’ with 1.59 ± 0.47 µm (Figure 13c,d). It was noted that the roughness amplitude achieved in the ‘rough’ proximal samples (S_a_ of 3.89 ± 0.14 µm) was greater than the ‘rough’ distal (1.77 ± 0.02 µm) and matched (1.76 ± 0.07 µm). The density of protruding peaks (S_pd_) above the core roughness amplitude (S_k_) was much greater in the ‘smooth’ samples compared to the ‘rough’ (5387 ± 515 mm^−2^ versus 1499 ± 92 mm^−2^, Figure 13e,f).

It was thought that the roughness amplitude after testing may be smaller than that of the as-manufactured surfaces. On average, samples presented only a slight reduction in Sa by 0.02 ± 0.03 µm (Figure 13a,b). The greatest difference was found in the ‘rough’ proximal samples by a small margin of 0.08 ± 0.17 µm. In contrast, S_k_ demonstrated an average increase of 0.03 ± 0.06 µm (Figure 13c,d). Unlike S_k_, the peak density (S_pd_) decreased in all test groups shown in Figure 13e,f. The ‘smooth’ samples decreased on average by 231 ± 114 mm^−2^ and the ‘rough’ by 30 ± 23 mm^−2^. Although the reduction in S_pd_ in the ‘smooth’ proximal samples was found to be statistically significant, the average magnitude of reduction was found to be comparable to the variation seen in the as manufactured surfaces.

**Figure 14:**
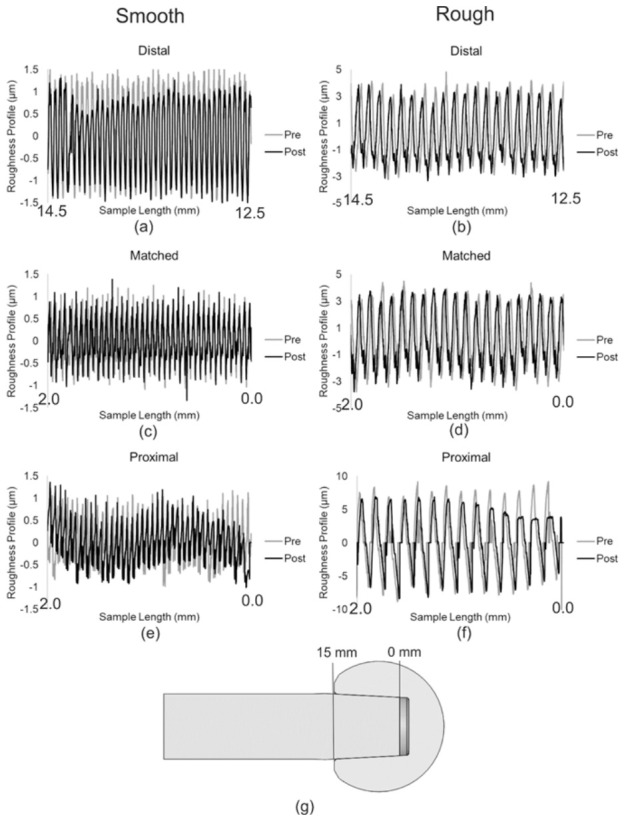
Example roughness profiles pre (grey) and post testing (black) of the (**a**) ‘smooth’ distal, (**b**) ‘rough’ distal, (**c**) ‘smooth’ matched, (**d**) ‘rough’ matched, (**e**) ‘smooth’ proximal and (**f**) ‘rough’ proximal samples. All profiles present a sample length of 2 mm long with the approximate location on the taper length indicted on each *x*-axis with respect to the (**g**) schematic.

Upon closer inspection of the scans, greater changes to the surfaces were seen more locally, especially in the distal and matched engagement groups (Figure 14). Figure 14a–f shows 2 mm traces of roughness profiles pre compared to post testing for each test group with respect to its position along the taper axis, as indicated in Figure 14g. Between 12.5 and 14.5 mm below the most proximal end of the male taper (Figure 14g), the ‘smooth’ distal samples (Figure 14a) presented an average reduction in S_a_ by 0.03 ± 0.01 µm. This compared to the ‘smooth’ matched (Figure 14c) and proximal (Figure 14e) samples that presented negligible differences in S_a_ (0.00 ± 0.01 µm) between 12.5 and 14.5 mm from the proximal end of the taper (Figure 14g). In contrast, the ‘smooth’ proximal samples presented the greatest reduction in S_a_ by 0.03 ± 0.01 µm at the proximal region of the interface (i.e., between 0 and 2 mm as indicated in Figure 14g) compared to the distal with 0.01 ± 0.01 µm and matched with 0.02 ± 0.01 µm. Comparing Figure 14c,e, differences to the ‘smooth’ matched appeared to be more uniform compared to the proximal.

Similar trends were seen between the different engagement groups in the ‘smooth’ samples was also reflected in the ‘rough’ samples. The ‘rough’ distal samples (Figure 14b) presented the greatest reduction in S_a_ between 12.5 and 14.5 mm from the proximal end of the male taper (Figure 14g) by 0.03 ± 0.02 µm. This compared to the ‘rough’ matched presenting an average reduction of 0.02 ± 0.02 µm and ‘rough’ proximal 0.01 ± 0.09 µm between 12.5 and 14.5 mm from the proximal end of the male taper. ‘Rough’ proximally engaged samples presented the greatest reduction in roughness amplitude at the proximal end of the taper (0 to 2 mm, Figure 14g), presenting an average reduction in S_a_ by 0.13 ± 0.03 µm. This compared to the distal and matched presented an average reduction in Sa by 0.01 ± 0.01 µm and 0.02 ± 0.01 µm, respectively.

**Figure 15:**
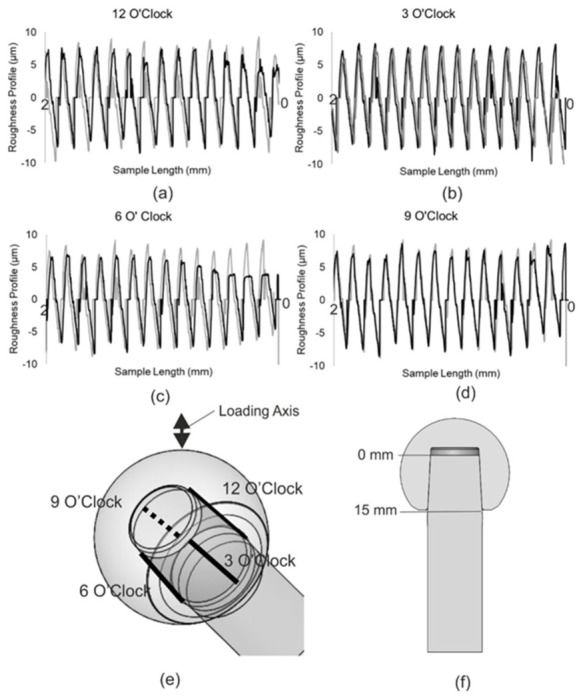
Example roughness profiles of a ‘rough’ proximal pre (grey) and post testing (black) at the (**a**) 12 o’clock, (**b**) 3 o’clock, (**c**) 6 o’clock and (**d**) 9 o’clock positions (**c**) relative to the taper geometry shown in (**e**) and taper axis shown in (**f**).

The more localised changes to the topography also appeared to vary with respect to the orientation around the taper axis. Figure 15 shows how roughness profiles around the taper axis varied for a ‘rough’ proximal sample. The greatest location of change was that in the 6 o’clock location, while the smallest change to the roughness profile appeared to occur in the 3 and 9 o’clock locations.

## 4. Discussion

It is well established that the degradation of modular tapers in vivo is complex, with an interdependent web of interactions that spans multiple disciplines [27], often making investigation into different design parameters difficult. Currently, there is a lack of common understanding of how surface topography and angular mismatch affect degradation at the taper junction. Therefore, this study conducted a systematic investigation into the fretting corrosion and motion response of samples that reflected the range of surface topography and angular mismatch seen clinically. A combination of short-term test methodologies, comparable to other researchers [53], were employed to systematically interrogate the role of modular taper design on the fretting corrosion and motion response.

### 4.1. The Role of Surface Roughness

Surface roughness was seen to have a significant effect on the fretting corrosion response of modular tapers. The ‘rough’ samples presented higher current measurements compared to the ‘smooth’, indicating a greater susceptibility to degradation via fretting corrosion. Although the effect of surface topography as a single design parameter on clinical performance is not universally agreed upon, retrieval studies generally support that an increased surface roughness amplitude contributes to taper degradation [31,32,33,34]. This was also seen in an experimental study by Panagiotidou et al. [37] that presented evidence of oxide disruption indicated by an increase in current upon the onset of dynamic loading with threaded ‘rough’ tapers, not shown by the non-threaded ‘smooth’ tapers. Results from this study indicate that all samples presented some evidence of passive oxide disruption (Figure 7). Average currents measured using the ‘rough’ samples presented between a five- and ten-fold increase compared to the ‘smooth’ of equivalent engagement. This was not reflected by a corresponding five- to ten-fold increase in motion, in either subsidence (Figure 10) or micro motion (Figure 12). Two key properties important to the electrochemical response that topography influences include contact stress at sites of interaction and surface area, both exposed to the solution and that experiencing abrasion. Assuming that increases in current from the baseline was due to the depassivation–repassivation of the passive oxide layer in line with Gilbert and Zhu [54], the ‘rough’ samples presented a greater proportion of abrasion and subsequent repassivation. The most probable explanation for this was differences in the contact mechanics at thread peaks.

In addition to differences in the magnitude of the measured current between the ‘rough’ and ‘smooth’ groups, the signal also differed. The ‘rough’ samples presented a more consistent ‘spike’ upon the onset of loading compared to the ‘smooth’ (Figure 7a versus Figure 7b). This ‘spike’ in current upon the onset of dynamic loading has been reported previously and found to correspond to subsidence events at the start of a new loading increment [53]. This study found that the ‘rough’ samples presented more gradual, step-wise increase in subsidence which was thought to contribute to the consistent spike in current upon the onset of each loading increment (Figure 7b and Figure 9b). The ‘smooth’ samples on the other hand presented evidence of discrete subsidence events, which were thought to be reflected in the less consistent spike in current (Figure 7b and Figure 9b). This was particularly poignant in the subsidence event shown in the ‘smooth’ matched sample in Figure 9a, and a corresponding spike and rapid decay in current upon the onset of the seventh increment shown in Figure 7a. The more gradual seating of tapers with an increased roughness has also been reported previously by Ouellette et al. [55] who found ‘ridged’ 9/10 tapers presented a ‘smooth’ seating plot while the ‘smooth’ 12/14 tapers presented a chattering/ratcheting (possible stick-slip) behaviour.

### 4.2. The Role of Angular Mismatch

**Figure 16:**
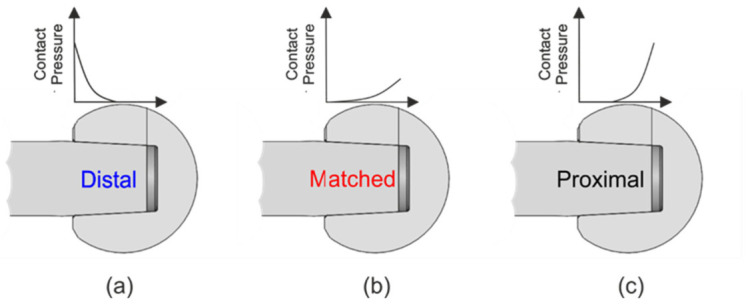
Schematic of contact pressure as a function of axial position within the interface for the (**a**) distal, (**b**) matched and (**c**) proximal angular mismatch groups.

There are a limited number of experimental studies that have undertaken a controlled systematic investigation into the effect of angular mismatch on motion and fretting corrosion. Computational studies unanimously report a reduction in relative motion with a reduced angular mismatch [38,39,40]. A logical hypothesis would therefore be that tapers with a reduced angular mismatch would result in less relative motion and therefore fretting corrosion. However, this study found that in both the ‘smooth’ and ‘rough’ samples, the matched engagement groups presented the greatest average current and charge compared to the distal or matched (Figure 8a,b,e,f). In contrast, the matched samples did not present the highest average micro motion. Although differences in micro motion between samples were comparable to the deviation seen between repeats, generally it was the proximal samples that presented the greatest pistoning micro motion (Figure 12a,b), and the distal samples that presented the greatest in the toggling directions (Figure 12d,e,g,f) and either the distal or proximal in the rotational direction (Figure 12j,k). One possible contributory explanation for the matched samples presenting the greatest fretting corrosion currents was a reduced interfacial contact pressure compared to the distal or proximal engagements. This is supported by surface analysis which found more uniform changes to the roughness profiles of the matched samples compared to the distal and proximal engagement samples that were more localised. Lower contact pressures resulting in more abrasion might seem counter-intuitive. However, within a fretting contact, higher pressures can sometimes be advantageous in terms of minimising sliding between the two surfaces by allowing motion to be accommodated by the contact stiffness [56]. Increased contact pressure has been shown to help minimise oxide layer disruption resulting in lower measured currents using electrochemical techniques in some instances [57]. Actual contact at the taper interface occurs at asperity junctions and the local contact pressure and degree of slip between the two surfaces will vary spatially due to number of variables including: implant geometry, surface roughness, local nano-mechanical properties and dynamic biomechanical loading. Within the matched samples, there was a higher probability of a greater portion of these asperities to experience gross slip for a greater proportion of oxide abrasion. Additionally, the matched samples presented a slight proximal mismatch (0.017 ± 0.004°), meaning that contact pressure is predicted to reduce towards the opening of the taper (Figure 16b). Therefore, asperity junctions with a higher probability of experiencing sliding occur closer to the taper opening, exposed to the electrolyte.

The distal engagement samples presented the lowest average current and charge transfer compared to the matched or proximal of equivalent surface topography (Figure 8a,b,e,f). Previous studies have suggested that a distal mismatch could help provide an effective seal [41,58]. Surface analysis in this study indicated that the distal samples did engage around the opening of the taper interface. One contributory reason to why the distal samples presented a reduced current response compared to the matched or proximal was the increasing trend in contact pressure towards to opening of the taper (Figure 16a). Contacting asperity junction exposed to the electrolyte would likely present a higher probability of being able to accommodate motion by a material response. However, the distal samples presented an increase in current above the baseline, suggesting that a complete seal was not achieved. In this study, when heads were assembled to 2 kN and subject to uniaxial loading up to peak forces of 4 kN, subsidence motions indicate that the heads were still seating. It remains to be seen whether the distal samples can support an effective seal.

**Figure 17:**
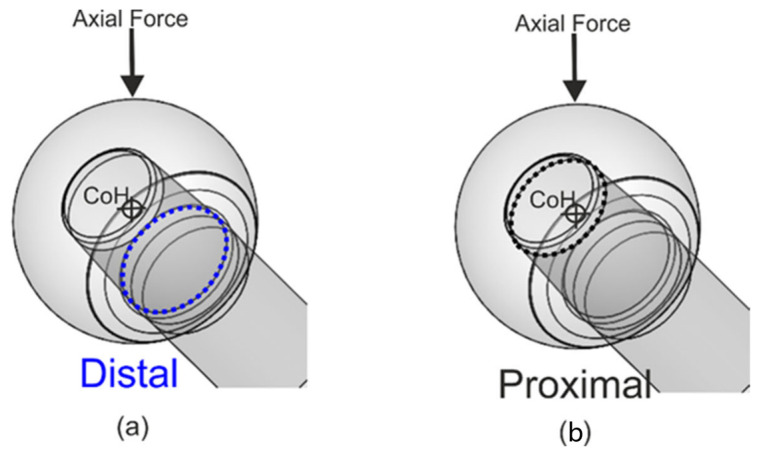
Schematic of engagement location with respect to the centre of rotation (CoF) for the (**a**) distal and (**b**) proximal samples.

The distal samples tended to present the least pistoning subsidence but the greatest off-axis and rotational subsidence motions (Figure 10a,b). Additionally, micro motion results indicate that they were able to resist pistoning micro motion to a similar degree as the matched (Figure 12a,b). Another mechanism that could have helped the distal samples resist oxide disruption compared to the proximal samples is an ability to resist pistoning micro motion. A possible explanation for why the distal samples can resist pistoning micro motion compared to a proximal engagement with a similar magnitude of angular mismatch (−0.090 ± 0.004° versus 0.120 ± 0.004°) could be the location of engagement with respect to the centre of the head (Figure 17a,b), where the distal engagement at the taper opening, away from the centre of the heads and loading axis, is better able to support pistoning micro motion after sufficient off-axis subsidence.

The proximally engaged samples represent the most severe and common engagements seen within clinically available THR system [23]. This study found that they presented a greater current response than the distal but smaller than the matched of equivalent surface topography (Figure 8). However, the proximal samples presented the greatest pistoning micro motion compared to the distal or match. It was also thought that this may have been reflected in the XZ direction, but the proximal samples presented the smallest average toggling XZ micro motion (Figure 12g,h). One explanation for this unexpected result was due to the use of a uniaxial loading profile. One hypothesis is that subject to more complex loading with a higher degree of dynamic off-axis loading may have further implications on a proximally engaged taper junction.

Results from this study indicate that design optimisations within the range of that seen in current clinically available THR systems can be made from an engineering perspective. When CoCrMo heads were assembled to 2 kN on stainless steel stems and dynamically loaded up to peak forces of 4 kN, smoother tapers with an increased angular mismatch can act to reduce the proportion of passive oxide layer disruption. However, this can have implications on taper mechanics in terms of subsidence and micro motion which have an unknown effect on the fretting corrosion response in the medium to long term.

Common practice within tapers used for industrial use is to relieve to the centre of the contact for a good fit without shaking due to contact at either end [18]. Within the taper junction in THR, this could offer a method of sufficient engagement around the taper opening while resisting off axis subsidence motions and pistoning micro motion. Future work will look at how these taper junctions perform under more complex loading and subject to greater assembly forces, a parameter consistently identified to have a significant effect on performance in vitro [55,59].

#### 4.2.1. Implications for Design and Verification

The present findings indicate that taper angular mismatch and male taper topography for HNSS-CoCr interfaces should be treated as coupled design and verification variables because they change both the electrochemical response of the junction and the dominant modes of micro-motion under load. Across the loading range investigated, angular engagement condition altered the charge transfer (a proxy for tribocorrosion propensity under the imposed electrochemical protocol) and the distribution of motion modes measured at the head–neck junction. In practical terms, this means that “low motion” should not be assumed to be synonymous with “low corrosion risk” and vice versa when specifying taper geometry and surface finish.

In this study, the engagement condition that was most benign in terms of cumulative charge was the configuration that promoted stable mechanical engagement under incremental uniaxial loading, whereas increased charge was observed when the local contact conditions promoted repeated disruption and reformation of the electrochemically active surface. Rougher, threaded male tapers produced a larger electrochemical response under equivalent loading, consistent with an increased tendency to disrupt passive films and sustain a higher faradaic contribution during fretting. Notably, the increase in electrochemical activity was not necessarily accompanied by a proportional increase in the magnitude of measured junction motion, reinforcing the need to quantify electrochemical and mechanical responses together when comparing design options.

From a mechanical stability perspective, the regime that minimised pistoning micromotion was the one that limited cyclic axial separation under load and preserved a more consistent engagement state. However, the data also show that some engagement conditions can shift the dominant motion away from axial pistoning and towards off-axis subsidence (toggling components), which is mechanically important because it implies eccentric contact, altered local stress concentrations, and a greater likelihood of off-axis loading at the taper interface. Such off-axis subsidence is clinically relevant because it may accelerate local fretting damage and facilitate a progressive change in contact mechanics even when axial micromotion appears relatively constrained.

These results suggest a pragmatic verification strategy for modular taper junctions. First, screening should include at least three representative angular engagement conditions (proximal-biased, matched, distal-biased) rather than relying on a single nominally “matched” configuration, since the present work demonstrates that engagement location can change both corrosion proxy metrics and motion mode balance. Second, surface topography should be appropriately quantified and bracketed (for example, a smooth and a threaded/rough condition) because roughness can amplify electrochemical activity even when measured micromotion changes are modest. Third, acceptance criteria should include both an electrochemical metric (for example, cumulative charge over a defined loading history) and a mechanical metric (e.g. motion, micromotion and subsidence) that separates axial pistoning from off-axis subsidence, as these capture different aspects of fretting-corrosion risk. Finally, the approach used here provides a basis for short-term preclinical screening that is sensitive to clinically plausible manufacturing tolerances and design choices and can be used to prioritise taper geometries and finishes for more resource-intensive whole-joint or multi-axis simulator testing.

#### 4.2.2. Limitations

This study examined a single, clinically relevant material pairing (CoCrMo head with high-nitrogen stainless steel trunnion), so the quantitative outcomes should not be assumed to transfer directly to other couples (for example, CoCr–Ti or ceramic–metal). The implication is not that taper design must be uniquely material-specific in a practical sense, but that material pairing can shift the balance between mechanical stability and electrochemical response because passive film behaviour, galvanic interactions, and fretting debris chemistry differ across alloys. A pragmatic interpretation is therefore to treat the design principles identified here (engagement location and topography influencing motion modes and charge transfer) as general, while requiring material-couple verification to confirm the magnitude and ranking for any given product system.

A key limitation this study presented compared to Mali and Gilbert’s [53] was the lack of concurrent electrochemical and motion measurements due to difficulties in calibrating the developed sensors used in this project within an aqueous environment. However, surface topography measurements of the samples before and after testing (Figure 13) indicate that any changes were marginal. This suggests that motion measurements conducted in series after disassembly and reassembly of these stochastic engagements can be used to help elucidate the fretting corrosion response.

The smaller rough cohort reduces statistical power and limits the extent to which the absolute magnitudes and between-group differences can be generalised. Accordingly, we interpret the findings primarily in terms of the direction of effects and their mechanistic plausibility, supported by within-specimen repeatability across the incremental loading steps, rather than as definitive population-level estimates. Future work will increase replicate numbers (guided by formal power calculations), include additional controls to better quantify between-manufacture variability, and test whether the same ranking holds across broader surface specifications.

## 5. Conclusions

In conclusion, results from this study show that surface and geometry design parameters of the taper interfaces in THR, in terms of fretting corrosion and motion, can be evaluated using short-term, multimodal, sensor-enabled preclinical test platforms. Key outcomes from this study include:

- Development and implementation of a motion sensing solution capable of capturing motion at the taper interface in three dimensions. Future work will involve measuring motion under more realistic biomechanical loading profiles compared to the uniaxial used currently.
- All the samples in this study presented some level of susceptibility to fretting corrosion indicating that design changes in terms of angular mismatch and surface topography cannot completely remove the issue of taper degradation in THR.
- The samples were still seating. Subsidence, during short-term tests, correlates with fretting corrosion measurements presenting limitations on the medium- to long-term insight afforded by this test methodology.
- Results indicate that CoCrMo heads coupled with a male HNSS taper and ‘rough’ threaded interface were more susceptible to fretting corrosion than a non-threaded ‘smooth’ modular taper interfaces.
- The distally engaged head stem couples, i.e., engagement around the opening of the taper interfaces, presented the lowest probable susceptibility to fretting corrosion compared to proximally engaged samples, i.e., engagements concentrated further within the interface.
- The most conforming tapers, the matched engagement samples (0.017 ± 0.004°), presented the highest net corrosion currents suggesting a higher susceptibility to fretting corrosion compared to tapers that present more severe angular mismatch.
- However, the matched samples also presented the least off-axis subsidence and some of the lowest levels of micro motion, suggesting possible implications under more complex loading with greater off-axis dynamic loading vectors.
- An experimental platform utilising clinically relevant samples was developed that enables streamlined assessment (versus current long-term ASTM/ISO testing) and further optimisation of medical device interfaces is proposed.

## Figures and Tables

**Table 1:** Details of angular mismatch and surface topography of the six different samples groups.

Sample Group	Angular Mismatch (°)	Male Taper Surface Finish
Smooth Distal 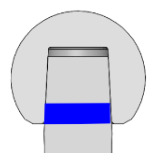	−0.090 ± 0.003	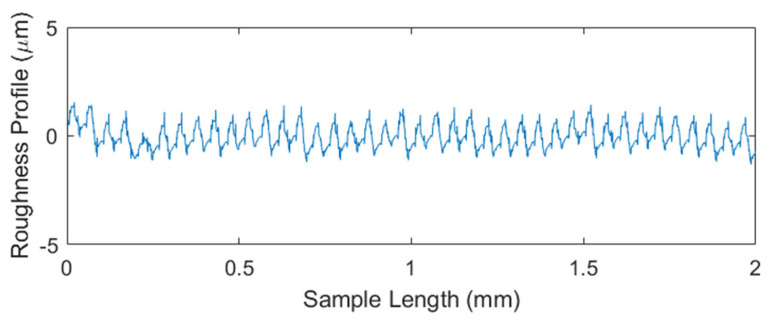
Smooth Matched 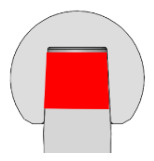	0.019 ± 0.003	
Smooth Proximal 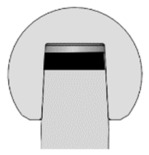	0.121 ± 0.013	
Rough Distal 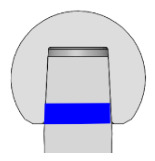	−0.088 ± 0.004	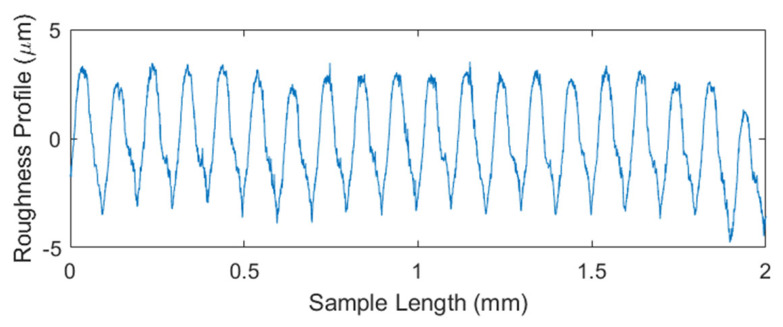
Rough Matched 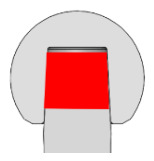	0.015 ± 0.004	
Rough Proximal 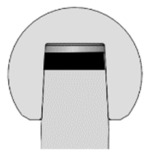	0.115 ± 0.004	

**Table 2:** Surface roughness parameters used to capture changes in surface topography due to testing and associated standards.

Roughness Parameter	Description	Applicable Standard or Paper
S_a_	Arithmetic mean of absolute values from the reference surface for a given sample length.	ISO 4287 [50]
S_k_	Depth of the roughness core profile. The roughness core profile being the roughness profile excluding protruding peaks and deep valleys.	ISO 13565 [51]
S_pd_	Number of peaks per area, i.e., peak density.	ISO 25178 [52]

## Data Availability

The datasets presented in this article are not readily available because due to IP restrictions. Requests to access the datasets should be directed to m.g.bryant@bham.ac.uk.

## Supplementary Materials

The following supporting information can be downloaded at: https://www.mdpi.com/article/10.3390/s26113571/s1, Figure S1: Schematic of motion sensing system including (a) coil board-target configuration, (b) distribution of coils on coil board and (c) coils-LDC-pc interface; Figure S2: (a) Schematic of the setup used for calibration, (b) calibration curves relating inductance to tar-get-coil distance and (c) residual displacements measured by the precision positioner stage from the calculated relationships relating inductance to displacement; Figure S3: Coordinate system for vector calculations for pistoning and toggling motions. Points A, B and C are three points on a plane and VAB and VAC are two vector which lie on the plane; Figure S4: C Motion captured for measurement of an equivalent monoblock sample. The dark grey represents the total motion and the lighter line following the ‘form’ of the motion represents subsidence.
