# Supplementary material for: How Angular Mismatch and Surface Topography in Modular Head–Stem Taper Junctions in Total Hip Replacements Affects Fretting-Corrosion and Motion Under Uni-Axial Loading
Source: Sensors (Basel). 2026 Jun 4;26(11):3571. doi: 10.3390/s26113571 (PMC13259156; doi:10.3390/s26113571)
Supplement: Supplementary file 1 [file sensors-26-03571-s001.zip › sensors-4314483-supplementary.pdf]

sensors

MDPI

Supplementary

How Angular Mismatch and Surface Topography in Modular Head–Stem Taper Junctions in Total Hip Replacements Affects Fretting-Corrosion and Motion Under Uni-Axial Loading

Abigail Wade [a], Andrew Robert Beadling [b], Dominic Jones [a], Danielle De Villiers [c], Jo Cullum [c], Simon Collins [c] and Michael George Bryant [b]

[a] University of Leeds, School of Mechanical Engineering, Institute of Functional Surfaces, Leeds, UK;
[b] School of Engineering, University of Birmingham, Brimingham, UK;
[c] MatOrtho®, Mole Business Park, Randalls Rd, Surrey, UK;

Motion Measurement Solution

Custom motion sensors were developed using the principle of eddy-current effect as the transducer mechanism. The eddy-current effect is a form of electromagnetic induction which may be utilised to measure the distance to a conductive object. A coil is excited by and AC current to generate an alternating magnetic field, which induces eddy currents in nearby conductive targets. These currents generate an opposing magnetic field, in accordance with Lenz's law, which cancels a part of the applied field and reduces the coil flux. This field coupling between the coil and conductive target acts to increase the coil's resistance and reduce the observed inductance. As the target moves closer to the coil, the increased alternating field increases the eddy-current density causing a further decrease in inductance. This change in inductance may be calibrated to the distance between the coil and target, giving a displacement sensor which is highly resistant to environmental effects [1].

To capture all types of motion at the taper interface, this study employed four sensing coils on a single custom PCB (P&M Services ltd, UK), allowing for four degrees of freedom measurement of the motion. The coils were mounted to the male component below the taper interface and aluminium targets were mounted on the femoral head, as shown in Figure S1a. Three coils were used to calculate the vector of a plane which allowed pistoning and toggling in two axes to be determined (Coils 1-3, Figure S1b). A fourth coil perpendicular to the other three was used to measure rotation (Coil 4, Figure S1b).

Texas Instruments (USA) coil and LDC design tools was were used to guide sensor design [2]. Each coil comprised two layers, each with 16 turns, 100 μm (or 4 mil) spacing between turn and 200 μm (8 mil) between layers, 100 μm trace width, an outer diameter of 10 mm and external capacitance of 330 pf. The coils were driven and monitored using a fully integrated, 4-channel inductance to digital converter chip (LDC 1614, Texas Instruments, US). The digital output of the LDC 1614 was then sent to a microcontroller (myRIO, National Instruments, US) via I2C protocol and inductance was recorded at 100 Hz. Figure S1c shows a schematic of how each component in the sensing system was connected. Raw inductances were then calculated and stored as a .txt file using a bespoke LabView (2019, National Instruments, US) program for export into Matlab (R2020a, MathWorks, US). A bespoke Matlab program was developed for the conversion of inductance to displacements and the different motions, i.e., pistoning, toggling and rotation.

 https://doi.org/10.3390/s26113571

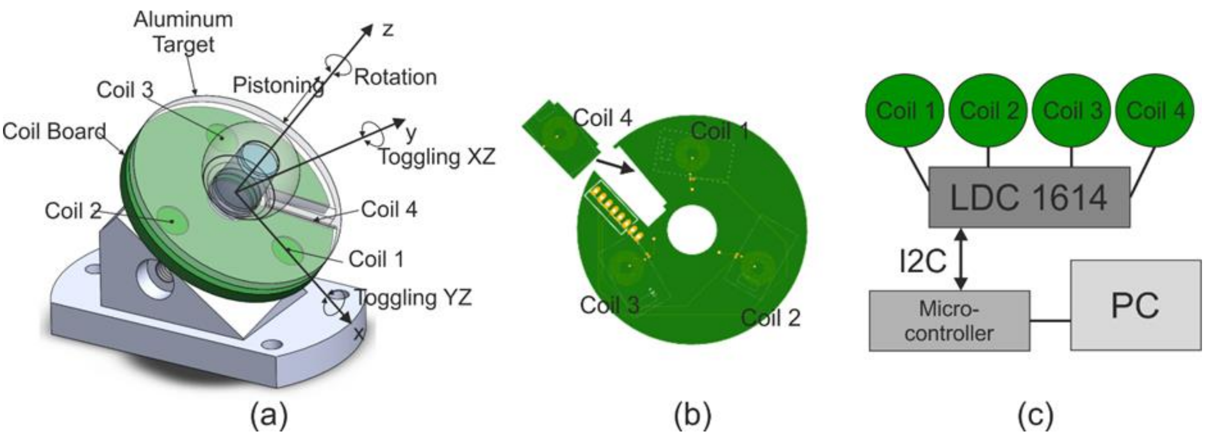

Figure S1: Schematic of motion sensing system including (a) coil board-target configuration, (b) distribution of coils on coil board and (c) coils-LDC-pc interface.

The sensors were calibrated using a piezo linear precision positioner stage (Mikro-Move, Physik Instruments, UK) accurate to 0.1 nm. Figure S2a shows a schematic of the setup used to calibrate Coils 1 to 3. This included fixing the aluminium target (Figure S2a) to the precision positioner stage and moving it relative to the coil board which was fixed to a spare male component. Coil 4 was calibrated separately before soldering into position. Calibration curves were determined across the full range of target–coil distances and shown in Figure S2b. Three separate sets of calibration data were then used to test the determined inductance-to-displacements relationships. Residuals from the three sets of data are shown in Figure S2c, which indicates that the sensing solution was found to be accurate to ± 0.5 μm.

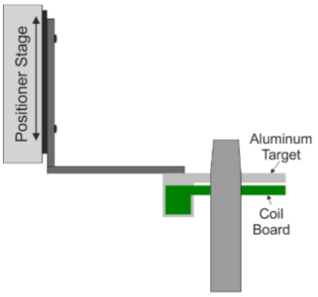

(a)

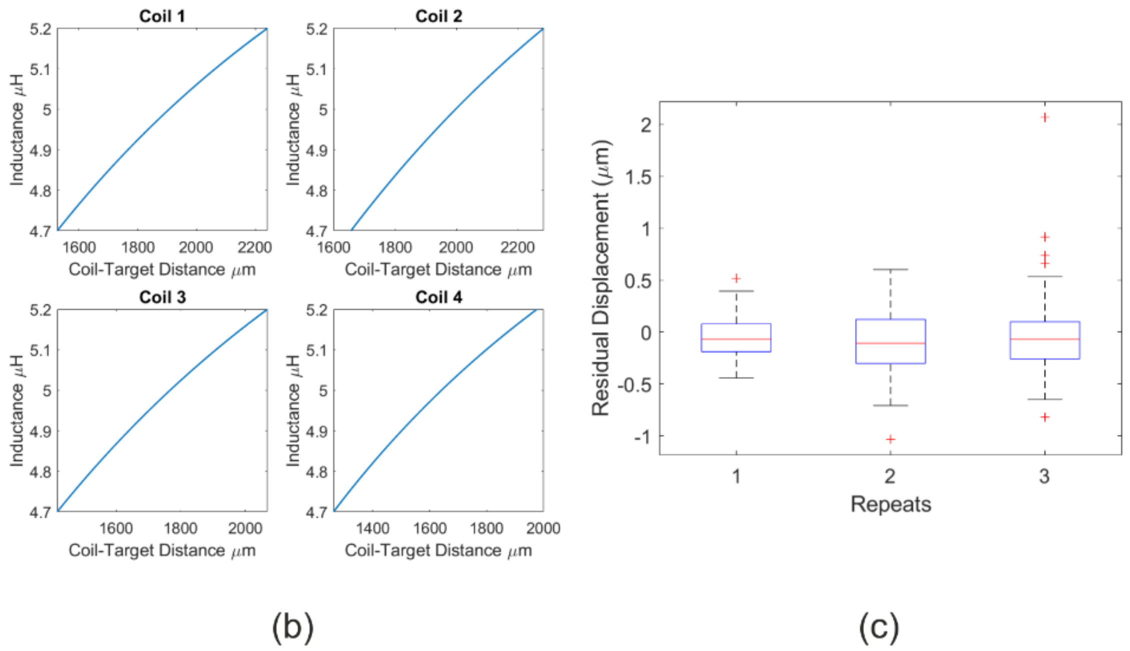

Figure S2: (a) Schematic of the setup used for calibration, (b) calibration curves relating inductance to target-coil distance and (c) residual displacements measured by the precision positioner stage from the calculated relationships relating inductance to displacement.

Four coils were used to capture all the different types of motion, including pistoning, toggling YX, toggling XZ and Rotation as shown in Figure S3. The x and y coordinates of the three points (A, B and C in Figure S3) were defined in the coordinate system shown in Figure S3 by the centre of each coil. The z coordinate was the measured target–coil distance.

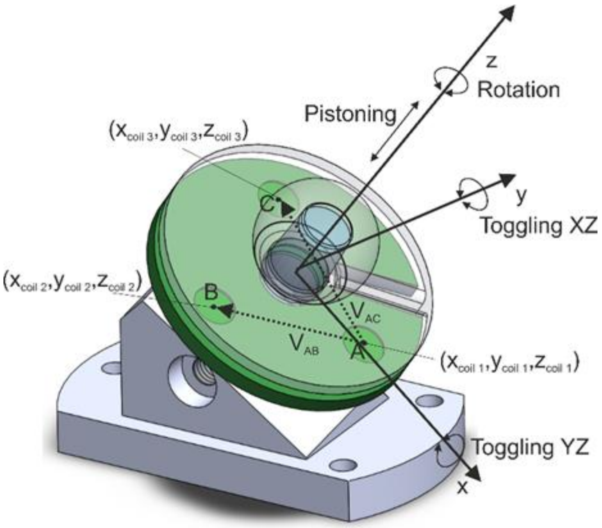

Figure S3. Coordinate system for vector calculations for pistoning and toggling motions. Points A, B and C are three points on a plane and V_{AB} and V_{AC} are two vector which lie on the plane.

Pistoning was determined by first calculating two vectors in the plane (VAB and VAC using three Points A, B and C, Figure S3). The second step was to determine the normal (n) to the plane by taking the cross product of these two vectors (Equation 1). Then, the perpendicular distance (d) from the origin to the plane was determined by taking the dot product of the normal and a point on the plane (Equation 2).

$$n = V_{AB}AB \times V_{AC}$$ *Equation 1*

$$d = A \; . \; n$$ *Equation 2*

Toggling YZ was found by first determining the angle the plane, at each sampling point, made with Y axis in the YZ plane, i.e., rotation about the x-axis (σ_x, Equation 3). Likewise, toggling XZ was found by determining the angle the plane, at each sampling point, made with X axis in the XZ plane, i.e., rotation about the y-axis (σ_y, Equation 4). Each rotation was converted to an equivalent displacement at the taper surface assuming a radius (r) of 7 mm using Equation 5 and Equation 6.

$$\sigma_x = 90 - cos^{-1}\left(\frac{n.\hat{j}}{|n|\;|\hat{j}|}\right)$$ *Equation 3*

$$\sigma_y = 90 - cos^{-1}\left(\frac{n.\hat{\imath}}{|n|\;|\hat{\imath}|}\right)$$ *Equation 4*

$$Toggling\,YZ = r\;tan(\beta_x)$$ *Equation 5*

$$Toggling\,XZ = r\;tan(\beta_y)$$ *Equation 6*

Rotation was calculated by use of a fourth coil perpendicular to Coils 1 to 3. Once the inductance was converted to a displacement, this was then used to calculate a rotation angle using a radius distance from the centre of Coil 4 to the z-axis. The displacement at the taper interface was then calculated as the arc length assuming a radius of 7 mm and the rotation angle derived from the coil. Each subsequent rotation displacement was subtracted from the first calculated value of rotation for relative position with respect to the start of the experiment.

All motions calculated from this study were subtracted from an equivalent monoblock to allow the removal of any elastic deformation measured from the test samples. The monoblock was a sample that was assembled to a high force of 8 kN and welded at the taper opening. Figure S4 shows the displacement of the four different motions measured on an equivalent monoblock, NB the recovery of displacement to that at the start of testing.

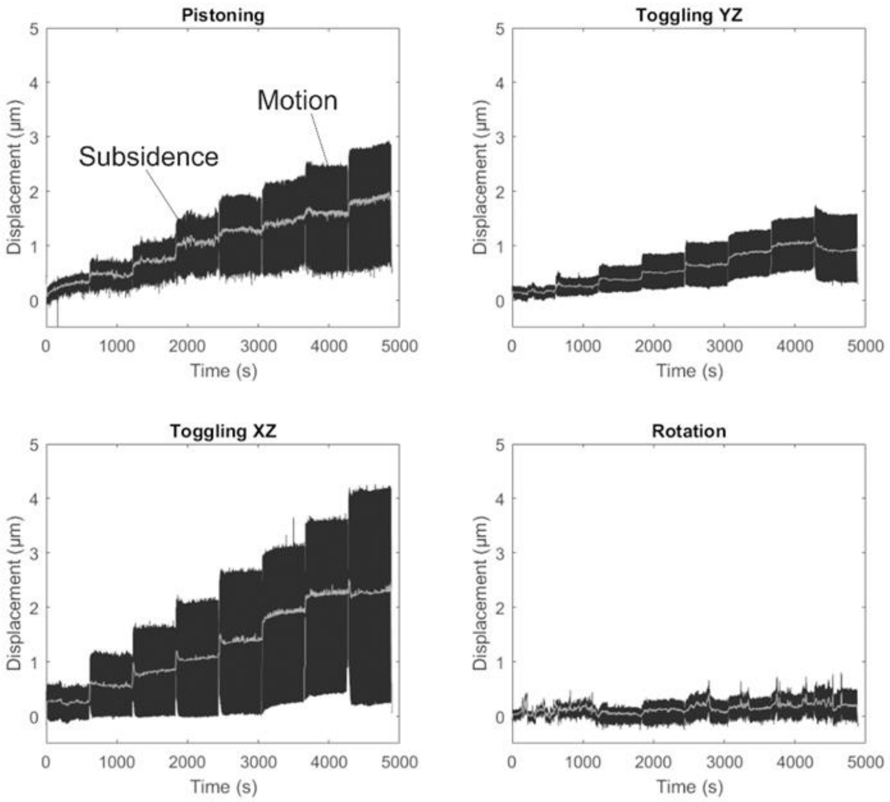

Figure S4. Motion captured for measurement of an equivalent monoblock sample. The dark grey represents the total motion and the lighter line following the 'form' of the motion represents subsidence.

References

1. Wang, H.; Feng, Z. Ultrastable and highly sensitive eddy current displacement sensor using self-temperature compensation. *Sens. Actuators A Phys*. **2013**, *203*, 362–368. https://doi.org/10.1016/j.sna.2013.09.016.
2. Oberhauser, C. *LDC Calculations Tool*; Texas Instruments: Dallas, TX, USA, 2016. Available online: https://e2e.ti.com/blogs_/b/analogwire/archive/2016/11/10/how-to-use-the-ldc-calculations-tool (accessed on 25 May 2026).
